# Supplementary material for: Development and Preliminary Evaluation of the Patient–Artificial Intelligence Rating Instrument
Source: medRxiv. 2026 Oct 22:2026.10.20.26366053. Preprint. [Version 1] doi: 10.64898/2026.10.20.26366053 (PMC13738826; doi:10.64898/2026.10.20.26366053)
Supplement: Supplementary file 1 [file media-1.pdf]

STROBE checklist

Cross-sectional study

Development and Preliminary Evaluation of the Patient–Artificial Intelligence Rating Instrument

Title, abstract, introduction, and methods

Item	Recommendation	Manuscript location / reporting
1(a)	Indicate the study's design with a commonly used term in the title or the abstract.	p. 2, Abstract: Design identifies a cross-sectional survey. Also stated on pp. 1 and 4.
1(b)	Provide in the abstract an informative and balanced summary of what was done and what was found.	pp. 2–3, Abstract.
2	Explain the scientific background and rationale for the investigation being reported.	pp. 5–6, Introduction.
3	State specific objectives, including any prespecified hypotheses.	p. 2, Abstract: Objective; p. 6, final Introduction paragraph. Instrument development and preliminary evaluation are the stated objectives.
4	Present key elements of study design early in the paper.	p. 2, Abstract: Design; pp. 6–9, Methods: Instrument development, Instrument, and Participants and setting.
5	Describe the setting, locations, and relevant dates, including periods of recruitment, exposure, follow-up, and data collection.	pp. 8–9, Participants and setting: Insight Panel and patient peers; anonymous online survey; ratings collected June 5–14, 2026. Recruitment dates are not separately specified.
6(a)	Give the eligibility criteria, and the sources and methods of selection of participants.	pp. 8–9, Participants and setting: adults aged ≥18 years with prior LLM use for personal health queries; email recruitment through the Insight Panel and peer contacts.
7	Clearly define all outcomes, exposures, predictors, potential confounders, and effect modifiers. Give diagnostic criteria, if applicable.	p. 8, Instrument: 12 quality items plus separately scored perceived-harm item; pp. 9–10, Statistical analysis; pp. 20–21, Tables 1–2. Exposure is participants' prior AI conversations (p. 2). No confounder-adjusted or effect-modification analyses.
8*	For each variable of interest, give sources of data and details of methods of assessment (measurement). Describe comparability of assessment methods if there is more than one group.	pp. 8–10, Instrument, Participants and setting, and Statistical analysis; p. 24, Figure 1. Self-administered survey, 5-point response scale, one response per conversation, identifiers linking repeat ratings. Table 1 footnote defines portal use (p. 20).

Methods: bias, sample size, and analysis

Item	Recommendation	Manuscript location / reporting
9	Describe any efforts to address potential sources of bias.	pp. 7–8, cognitive interviews to refine item interpretation; p. 9, anonymous survey; pp. 9–10, analyses addressing repeated ratings; p. 14, selection of engaged AI users and elevated ratings acknowledged.
10	Explain how the study size was arrived at.	p. 9, Statistical analysis: no formal sample-size calculation; all eligible panel members invited; size determined by panel size and response during the collection window.
11	Explain how quantitative variables were handled in the analyses. If applicable, describe which groupings were chosen and why.	pp. 8–10, Instrument and Statistical analysis: 1–5 item responses, 12-item quality scale, harm summarized separately, first-rating and participant-mean analyses. Table 1 (p. 20) identifies demographic categories and digital-literacy grouping.
12(a)	Describe all statistical methods, including those used to control for confounding.	pp. 9–10, Statistical analysis: Cronbach α and Feldt confidence intervals; clustering sensitivity analyses; random-effects ANOVA and ICC(1); item statistics, covariance/correlation matrices, and descriptive harm distributions. No confounder-adjusted analyses.
12(b)	Describe any methods used to examine subgroups and interactions.	Not applicable: no subgroup comparisons or interaction tests are reported. Repeat-rater sensitivity analyses are described under item 12(e).
12(c)	Explain how missing data were addressed.	p. 10, Statistical analysis and Sample: complete PAIR ratings only; demographics and AI-use data use available responses; no imputation. Four incomplete survey submissions excluded. Table 1 footnote (p. 20) gives demographic and AI-use denominators.
12(d)	If applicable, describe analytical methods taking account of sampling strategy.	pp. 9–10, Statistical analysis: repeated ratings addressed by first-rating and participant-mean sensitivity analyses and variance partitioning. Convenience sampling is described on pp. 2 and 8–9; no probability-sampling weights are reported.
12(e)	Describe any sensitivity analyses.	p. 10, Statistical analysis: Cronbach α recalculated using each participant's first chronological rating and participant-mean item profiles. Results on p. 11.

Results

Item	Recommendation	Manuscript location / reporting
13(a)*	Report numbers of individuals at each stage of study, eg numbers potentially eligible, examined for eligibility, confirmed eligible, included in the study, completing follow-up, and analysed.	p. 10, Sample: 14 participants contributed 46 complete ratings; 4 additional incomplete survey submissions were excluded. Five participants rated one conversation and nine rated multiple conversations.
13(b)*	Give reasons for non-participation at each stage.	p. 10, Sample: incompleteness is the stated reason for excluding 4 survey submissions.
13(c)*	Consider use of a flow diagram.	No flow diagram is provided.
14(a)*	Give characteristics of study participants (eg demographic, clinical, social) and information on exposures and potential confounders.	p. 20, Table 1: participant characteristics; p. 21, Table 2, and p. 11, Use types: conversation purposes and information entered into AI tools.
14(b)*	Indicate number of participants with missing data for each variable of interest.	p. 10, Sample: demographics available for 13 of 14 participants; all 46 analyzed ratings complete on all 13 items.
15*	Report numbers of outcome events or summary measures.	pp. 11–12, Internal consistency, Item-level performance, Inter-item covariance structure, and Harm item; pp. 22–23, Tables 3–4.
16(a)	Give unadjusted estimates and, if applicable, confounder-adjusted estimates and their precision (eg, 95% confidence interval). Make clear which confounders were adjusted for and why they were included.	p. 11: α estimates and 95% CIs for all ratings, first ratings, and the 13-item set; participant-mean α and ICC(1) are point estimates without CIs. Item estimates in Table 3 (p. 22).
16(b)	Report category boundaries when continuous variables were categorized.	p. 20, Table 1: education categories and digital-literacy grouping (agree or strongly agree) are identified. Age is summarized continuously as median and IQR
16(c)	If relevant, consider translating estimates of relative risk into absolute risk for a meaningful time period.	Not applicable: no relative-risk estimates are reported.
17	Report other analyses done, eg analyses of subgroups and interactions, and sensitivity analyses.	p. 11: first-rating and participant-mean α, repeat-rater variance partitioning, and α including harm; p. 12: participant-level harm distribution and covariance structure; Table 4 (p. 23).

Discussion and funding

Item	Recommendation	Manuscript location / reporting
18	Summarise key results with reference to study objectives.	pp. 12–13, opening Discussion paragraphs.
19	Discuss limitations of the study, taking into account sources of potential bias or imprecision. Discuss both direction and magnitude of any potential bias.	p. 13: clustering estimate based on only nine repeat raters needs confirmation. p. 14, Limitations: small, highly engaged sample; likely elevated item means and restricted range; no criterion/construct-validity claims. Bias magnitude is not formally quantified.
20	Give a cautious overall interpretation of results considering objectives, limitations, multiplicity of analyses, results from similar studies, and other relevant evidence.	pp. 12–14, Discussion, Limitations, and Conclusions: preliminary internal consistency and conversation-level variation; complementary role alongside clinician evaluation; broader validation needed.
21	Discuss the generalisability (external validity) of the study results.	p. 14, Limitations: engaged AI-using patient advocates, restricted response range, and need for broader patient and language representation.
22	Give the source of funding and the role of the funders for the present study and, if applicable, for the original study on which the present article is based.	pp. 14–15, Article Information: Funding/Support and Role of the Funder/Sponsor. NLM/NIH award R01LM014689; no funder role in study conduct, reporting, or submission decision.

* Where applicable, report exposed and unexposed groups separately. No such comparison is reported in this study. Cohort- and case-control-only checklist items are not applicable to this cross-sectional design.

STROBE: Strengthening the Reporting of Observational Studies in Epidemiology. Checklist recommendations: www.strobe-statement.org.
